# Florigen and its homologs of FT/CETS/PEBP/RKIP/YbhB family may be the enzymes of small molecule metabolism: review of the evidence

**DOI:** 10.1186/s12870-022-03432-z

**Published:** 2022-01-27

**Authors:** Olga Tsoy, Arcady Mushegian

**Affiliations:** 1 Chair of Experimental Bioinformatics, TUM School of Life Sciences Weihenstephan, Technical University of Munich (TUM), 3, Maximus-von-Imhof-Forum, 85354 Freising, Germany; 2 Current address: Chair of Computational Systems Biology, University of Hamburg, Notkestrasse, 9, 22607 Hamburg, Germany; 3 Molecular and Cellular Biology Division, National Science Foundation, 2415 Eisenhower Avenue, Alexandria, Virginia, 22314 USA; 4 Clare Hall College, University of Cambridge, Cambridge, CB3 9AL UK

**Keywords:** Florigen, Flowering, Phosphatidylethanolamine-binding protein, Raf-kinase interacting protein, YbhB, Tautomycin

## Abstract

**Background:**

Flowering signals are sensed in plant leaves and transmitted to the shoot apical meristems, where the formation of flowers is initiated. Searches for a diffusible hormone-like signaling entity (“florigen”) went on for many decades, until a product of plant gene *FT* was identified as the key component of florigen in the 1990s, based on the analysis of mutants, genetic complementation evidence, and protein and RNA localization studies. Sequence homologs of FT protein are found throughout prokaryotes and eukaryotes; some eukaryotic family members appear to bind phospholipids or interact with the components of the signal transduction cascades. Most FT homologs are known to share a constellation of five charged residues, three of which, i.e., two histidines and an aspartic acid, are located at the rim of a well-defined cavity on the protein surface.

**Results:**

We studied molecular features of the FT homologs in prokaryotes and analyzed their genome context, to find tentative evidence connecting the bacterial FT homologs with small molecule metabolism, often involving substrates that contain sugar or ribonucleoside moieties. We argue that the unifying feature of this protein family, i.e., a set of charged residues conserved at the sequence and structural levels, is more likely to be an enzymatic active center than a catalytically inert ligand-binding site.

**Conclusions:**

We propose that most of FT-related proteins are enzymes operating on small diffusible molecules. Those metabolites may constitute an overlooked essential ingredient of the florigen signal.

**Supplementary Information:**

The online version contains supplementary material available at 10.1186/s12870-022-03432-z.

## Background

Flower production in plants occurs in response to the environmental cues – most importantly, changes in the day length. The role of photoperiodicity in all living forms from bacteria to higher eukaryotes is well established, and numerous studies have shown that the perception of the photoperiodic signal in plants occurs primarily in the leaves. Flowers, however, are formed mostly by shoot apical meristems (SAM) that are typically shielded from direct light, and the mechanisms conveying the flowering signal from leaves to SAMs have been a matter of speculation and investigation for most of the twentieth century.

Plant physiologists have established, by the 1930s, that angiosperms generally fall in three categories, i.e., long-day plants, in which blooming is turned on by day lengthening - night shortening; short-day plants, which initiate blooming upon day shortening - night lengthening; and day-neutral plants, which bloom in response to the cues other than day length [32, 58]. Experiments on floral induction in partially shaded plants and in grafts between light-induced and uninduced plants suggested the existence of a diffusible substance promoting floral transition; the idea may have been first proposed by Julius Sachs [95], but was consolidated in the modern form by M. Chailakhyan (1902–1991), who proposed the term “florigen” as the name for the flower-inducing chemical entity [21]. The review of early research on the photoperiodic signal sensing in leaves can be found in Zeevaart [123] and Kobayashi and Weigel [60], and an account of Chailakhyan’s remarkable life and scientific work has been given in Romanov [93].

The transfer of a flowering signal from leaves to the shoot apex has been studied over the years in many species of flowering plants, and general similarity of its properties to those of small molecules was noted; for example, the florigen fraction appeared to move in phloem at the rate comparable to that of other plant assimilates [54]. It has been established also that grafts and extracts of induced plants could transfer flowering ability not only to the uninduced vegetatively developing plants of the same species, but sometimes also to other species or genera, suggesting the evolutionary conservation of the signaling pathways [122].

Despite general acceptance of the idea of florigen as a conserved hormone-like substance, the attempts at the isolation and chemical characterization of the responsible entity yielded no results for several decades. A review of state of the affairs in 1976 listed the factors that have been tested unsuccessfully for the ability to induce floral transition; among the failed candidates there were sugars, amino acids, sterols, gibberellins, salicylic acid, ethylene, cytokinins, the photosynthetic capacity, initiation of protein synthesis, and others [122]. Several hypotheses were put forward to explain the difficulties of identifying florigen: perhaps it was not one molecule but several distinct hormones or metabolites, acting jointly in a specific succession, or as a mix with specific ratio of components; or, possibly, the identity of florigen was masked by simultaneous presence of flowering inhibitors in the same samples [63]; or, maybe, flowering was caused by propagating a signal that spread from leaves to meristems not in chemical, but in electric form, such as plasma membrane potential [84].

Despite all the work to test these and other hypotheses, there was little progress in biochemical characterization of florigen until early 1990s, when the search for a flower-inducing activity started employing the tools of molecular biology. For example, in what may have been the last published study by Chailakhyan, a radiolabeled protein band of ~ 27 kDa was observed in the induced, but not in naïve, leaves of *Rudbeckia*, followed by accumulation of a similar-sized band in the shoot apex tissues ([76]; the work is largely unavailable to the Western reader because of the temporary interruption of translation and indexing of Russian-language journals upon the collapse of the Soviet Union). Around the same time, it has been shown [107] that aqueous extracts of *Lemna*, *Pharbitis* and *Brassica* contained a flower-inducing fraction dominated by a 20–30 kDa protein band; the authors noted, however, that the florigen activity was unaffected by proteinase K digestion that removed the protein, perhaps suggesting a role for an associated small molecule.

Nearly simultaneously, the results of genetic screens for *Arabidopsis thaliana* mutants with late flowering phenotypes were published [61]. This was a watershed moment in the studies of the molecular determinants of flowering initiation, and important discoveries ensued in the following three decades have been. The state of the knowledge may be outlined as follows (summarized from the following reviews – [6, 47, 49, 56, 69], which can be consulted for additional details and for the timeline).

Flowering in Arabidopsis is long day-dependent, and is enabled through the circadian clock-controlled transcriptional co-regulator *CONSTANS (CO)* and its target *FLOWERING LOCUS T (FT).* The protein product of the latter gene has emerged as the integrator of the environmental inputs, relaying these signals into the gene regulatory networks that control flowering. The FT protein of 19.8 kDa (176 amino acids) is produced in the phloem companion cells of the leaves, enters phloem sieve elements and is transported from leaves to the base of shoot apical meristem, where it has been detected experimentally. Considerable evidence exists that the *FT* gene is expressed in SAM mostly or only in its basal portion, and that the FT protein may move from cell to cell in plants. This is a key set of properties expected of florigen. A strong genetic and transgenic evidence suggests that FT is required for activating the expression of floral meristem identity and flowering time genes, such as MADS-box transcription factors SUPPRESSOR OF OVEREXPRESSION OF CONSTANS 1 (SOC1), FRUITFULL (FUL/AGL8) and AGL24, and ultimately the master regulator of flower development LEAFY (LFY). Orthologs of FT, such as SINGLE FLOWER TRUSS (SFT) in tomato, HEADING DATE 3a (HD3a) in rice, and their counterparts in other plant species (sometimes called CETS proteins in the plant context, after the names of several better-studied representatives from *Antirrhinum*, *Arabidopsis* and *Lycopersicon*) share many of these properties with FT, with some variation in the precise wiring of the transcriptional networks.

The homologs of *FT*, found in varying numbers in all examined angiosperm species, also tend to be involved in flowering control, many of them acting synergistically, redundantly or antagonistically with *FT;* perhaps the best-studied antagonistic pair is Arabidopsis gene *FT* and its paralogous gene *TFL1* and the counterparts of those two genes in other flowering plants similarly have opposing effects on plant development [4, 59, 62, 68, 73, 89, 112, 119]. FT and its homologs have been also implicated in other aspects of plant organogenesis, such as seed germination in bamboo and tuber formation in potato, as well as in a growing variety of physiological processes, such as vacuolar sorting, stomata opening, and sink-source regulation [3, 24, 26, 49, 52, 55, 87, 103].

Despite all the effort to pinpoint its precise location, FT protein has not been unambiguously detected in phloem-free tips of SAM in any plant, only in the basal portions of the meristem zones. Protein localization studies at a single-cell resolution in vivo remain challenging, and it is not clear whether FT actually reaches the cells where the meristem identity genes are expressed. The argument that FT does just that – as opposed, for example, to activating an additional low molecular weight messenger – has been made in the literature, but the evidence is indirect, showing for example that some engineered fusions of FT to green fluorescent protein-based reporters are retained in the bottom half of the SAM zone, and in those cases they do not complement the *ft* recessive mutants [25]. A study in rice [108] has made a more direct claim, i.e., that Hd3a, the rice protein orthologous to FT, does reach the tip of the SAM to induce flowering. However, their Fig. 3E, F, M, and N, cited as the key supporting evidence, does not seem to show the protein reporter activity at the tip, where it is supposed to be – only in the bottom halves of the SAM zone, just like in the case of Fig. 2G in Corbesier et al. [25]. More recent analysis used a sensitive fluorescent assay to report that FT is found in the basal as well as the apical part of SAM [2], though their Fig. 2 again shows a fluorescence-negative zone at the extreme apex. In fact, it has been hypothesized that, contrary to a naïve expectation, the restriction of FT to the basal portions of SAMs may be a tightly regulated, TFL1-mediated event, actually beneficial for maintaining the supply of undifferentiated stem cells in the more apical parts [13, 120]. Be it as it may, it remains possible that FT is but one component of the flower induction signal, and that a small molecule, possibly activated by or acting synergistically with FT, is also involved [6, 68].

Another gene of Arabidopsis known to be involved in flowering control, *FD*, has been identified in a robust genetic screen as a recessive suppressor of *FT*; the ability of overexpressed FT to induce precocious flowering is impaired in plants with the lowered production of FD [1]. The *FD* gene encodes a protein from the bZIP-type transcription factor family, which physically interacts with the FT protein in yeast two-hybrid system, in the bi-molecular fluorescence complementation assays in vivo, and apparently in the affinity-purified protein complexes [1, 118]. In rice, the FT ortholog HD3a does not interact with the bZIP factors directly, but co-precipitates with the FD ortholog OsFD1 in a tripartite complex that also includes a scaffolding 14–3-3 protein [109]. Largely on the basis of this evidence, FT has been assigned the molecular function of a transcriptional co-activator, proposed to form a putative multisubunit complex that binds to the regulatory regions and controls the expression of the flowering identity genes. A corroboration of these protein interaction experiments is provided by the analysis of gene expression and ChIP-Seq data, which reveal that tagged FT binds, apparently mostly in the FD-dependent manner, to the DNA regions located near many of the genes involved in flowering control [124].

The emerging picture of the FT role in transcriptional control of gene expression is complicated by the subcellular localization studies of FT and its homologs. FT appears to be transported into the nucleus in a FD-dependent manner, but remains largely cytoplasmic in *fd* plants [2]. On the other hand, its best-studied paralog TFL1 in Arabidopsis has been localized to plasma membrane, tonoplast, and dense vesicles in situ, and to the membranes of fractionated protoplasts, with direct implications in protein trafficking to the storage vacuoles [103], whereas the ortholog of TF in potato, StSP6A, has also been seen in association with membranes [3].

Analysis of the amino acid sequence has shown that FT protein belongs to a widely conserved sequence family, members of which are encoded by genomes of many bacteria, archaea and nearly all eukaryotes. The founding member of the family, isolated from bovine brain, is a hydrophilic, cytoplasmic protein that can bind in vitro to many low molecular weight compounds of different chemical structure, including certain phospholipids [15]. The name phosphatidylethanolamine-binding protein (PEBP) became attached to the family, though functional relevance of phosphatidylethanolamine binding has not been demonstrated for any homolog of this protein in any species. The genes or protein products of FT/PEBP family turn up in a surprisingly large variety of genetic screens and binding assays. This has resulted in a long list of putative properties assigned to these proteins, including, in addition to in vitro phospholipid binding in plant and animal homologs, also inhibition of carboxypeptidase Y in vitro and regulation of Ras GTPase in vivo by the yeast homolog Tfs1 [16, 36]; inhibition of Raf-1 kinase in mammals – hence an alternative name of the protein family, Raf-Kinase Inhibitor Protein, or RKIP [121]; suppression of trans-epithelial migration of the mammalian host neutrophiles by YbcL, a PEBP homolog from uropathogenic *E.coli* [64]; and an uncharacterized role in the modification of polyketide chains in *Streptomyces* [66, 67]. In a continuation of the theme of diverse cellular locations in the family members, one of the FT co-orthologs in mammalian cells, PEBP1, directly interacts with some isoforms of membrane-localized 15-lipoxygenase and controls their function [115], whereas at least three animal and one bacterial homolog, i.e., mammalian PEBP4, a predicted odorant-binding protein in fruit fly, a putative venom gland constituent in gall wasp, and aforementioned YbcL, are either known or predicted to be secreted extracellularly [19, 40, 64, 86].

**Fig. 1:**
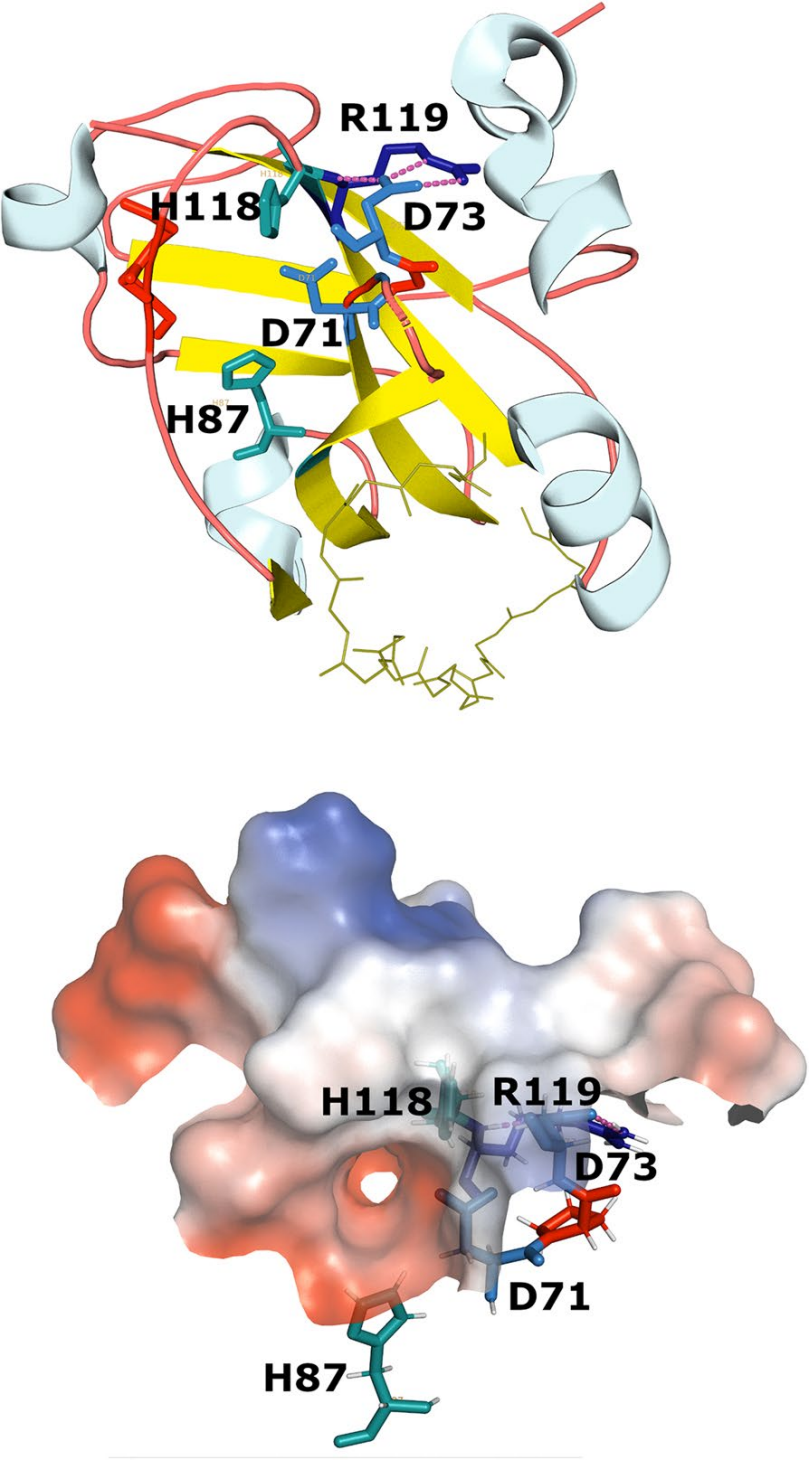
Spatial organization of the conserved residues forming putative catalytic center in FT protein from *Arabidopsis thaliana* (PDB ID 1wkp). The three-dimensional structures of proteins were visualized, and their approximate charge-smoothed electrostatic surface representations were generated using the open-source PyMOL environment (Schrödinger LLC; SciCrunch RRID SCR_000305), installed from source using homebrew on the MacOS High Sierra 10.13.6. Top panel: strands are rendered in yellow, helices in light blue, and conserved residues involved in forming the putative enzyme active center are labeled and colored as follows: blue, two conserved aspartates; cyan, two conserved histidines; dark blue, conserved arginine; red, frequently conserved prolines. The loop between strands 3 and 4 is reduced to a short broken wire to improve the visibility of the active center, and the loop that is a major determinant of the differential activity of FT and its paralog TFL1 [4] is rendered as sticks. Bottom panel: The putative enzyme active center rendered as a surface. A rough calculation of the surface electrostatic properties in the vacuum was performed using PyMOL function generate → vacuum electrostatics. The shades of red and blue indicate, respectively, negative and positive charges. The conserved residues involved in forming the putative enzyme active center are labeled, and their colors are the same as in the top panel

Structural studies of FT/PEBP-like proteins from diverse species of bacteria and eukaryotes, conducted by X-ray crystallography and nuclear magnetic resonance approaches [8, 11, 12, 29, 37, 77, 90, 99, 100, 110] have revealed a distinct spatial fold, with a structural core dominated by two beta-sheets. Structurally, the fold superficially resembles an immunoglobulin-like arrangement but is not specifically related to any other fold (entry 11.1.7 in the ECOD database; Cheng et al., [23]). A prominent structural feature of this family is an evolutionary conserved depression on the surface of the molecule; the rim of the cavity is lined by three of the residues conserved in the entire family, i.e., an aspartic acid located at the C-terminus of strand 3 (in the FT protein of Arabidopsis, it is denoted D71 [42]) and two histidines found at the N-termini of strands 4 and 6, denoted H87 and H118. The quintet of the most conserved residues is completed by another aspartic acid close to the first one (D73), and an arginine that adjoins the second conserved histidine (R119) and bonds via a salt bridge with D73, forming a “shoulder” next to the cavity [99]. The spatial configuration of these residues and select other conserved features in Arobidopsis FT protein are rendered in Fig. 1.

In the known crystal structures, the cavity sometimes accommodates anions included in the media, but it is neither hydrophobic nor large enough to bind lipids. On the other hand, several sites suitable for binding hydrophobic ligands have been inferred on the molecule by in silico studies, but those sites tend to be located in the least conserved regions of the molecule [4, 39, 83, 92, 94, 101]. Curiously, the site of interaction between FT and FD in *A.thaliana* has not been structurally characterized.

In this work, we highlight the patterns of sequence and structure conservation in the family and present the results of the analysis of genomic context of the FT/PEBP proteins in prokaryotes. We argue that the total evidence is best compatible with the idea that these proteins are not only (or even perhaps not at all) transcriptional co-activators, but enzymes involved in production, conjugation, or removal of low molecular weight ligands.

### Survey and summary of the computational evidence

#### Sequence conservation in the FT homologs throughout the Tree of Life suggests shared ancestry and common molecular function

We have collected the homologs of plant FT proteins by searching the NCBI NR protein database, or databases restricted by taxon (e.g., viruses), focusing on completely sequenced genomes. The database searches were done using PSI-BLAST program with standard settings [5], mostly in June 2021. We also consulted the NCBI COG resource that annotates conserved orthologous genes in bacteria and archaea [31]. In the following, unless specified otherwise, we refer to the prokaryotic FT homologs as YbhB proteins, after the chromosomal locus in *E.coli* that has orthologs in many other bacteria, and use some of the FT/CETS/PEBP/RKIP name aliases for eukaryotic homologs.

Complete genomes of the unicellular and multicellular eukaryotes tend to encode at least one, or commonly more than one, FT homolog; a rare exception are some parasitic eukaryotes with reduced genomes, such as microsporidia, which do not appear to have genes from this family. YbhB genes (NCBI COG1881) are found in almost all major lineages of bacteria and archaea. Among the clades of archaea, only methanogenic *Euryarchaeota* appear to lack the YbhB homologs*,* and among bacteria, only the phylae *Firmicutes*, *Mollicutes* and the order *Spirochaetales* mostly contain species that are YbhB-free. In other clades of bacteria and archaea, between 30 and 80% of all species encode YbhB-family proteins. All told, 782 copies of YbhB-family proteins are found in 594 bacterial and archaeal genomes out of the 1309 genomes in the 2020 release of the COG database [31]. YbhB homologs are also encoded by the genomes of many giant DNA viruses from the class *Megaviricetes* and from some related lineages.

**Fig. 2:**
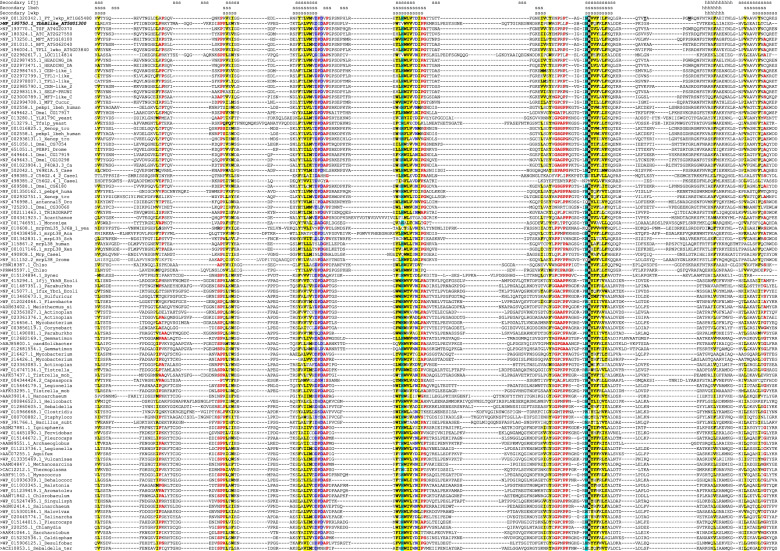
Multiple sequence alignment of select members of FT/CETS/PEBP/RKIP/YbhB family. Sequence identifiers in GenBank or PDB are shown before each sequence. For the arabidopsis proteins, gene product names and AGI locus codes are also shown. In the Secondary structure lines above the alignment, s stands for a beta-strand and h stands for an alpha-helix. Conserved hydrophobic residues (I, L, M, V, F, Y, W) are indicated by yellow shading, conserved small-side-chain or turn/kink-prone residues (A, G, S, P) are indicated by bold red type, and the conserved constellation of charged residues discussed in the text are marked as follows: gray-shaded blue type, two conserved aspartates; cyan shading, two conserved histidines; and black-shaded white type, conserved arginine. In the sequence XP_024370999.1, the bold underlined X marks the position of a low-complexity sequence insertion that most likely represents a falsely predicted exon

**Fig. 3:**
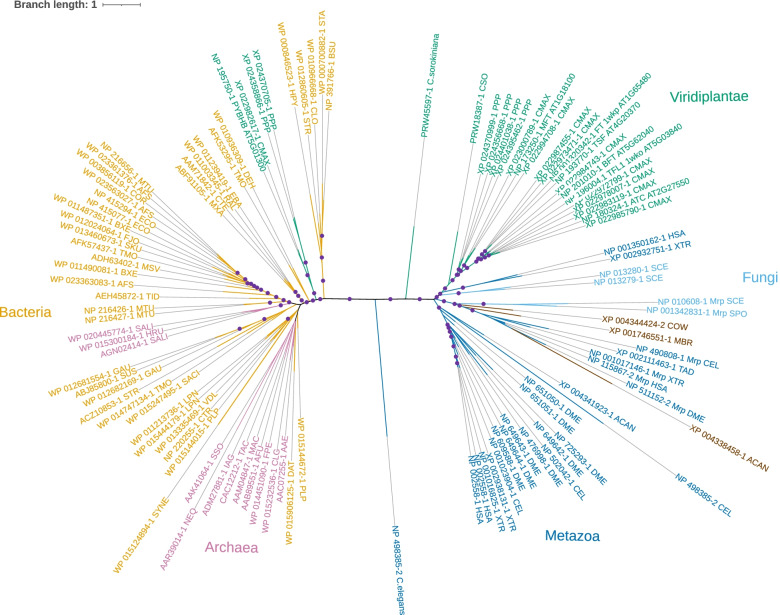
Phylogenetic tree of the FT/CETS/PEBP/RKIP/YbhB family. Bacterial sequences and branches leading to them are shown by the tan color; archaeal sequences are in lilac, fungal sequences are in light blue, metazoa are in dark blue, protists are in black and plants are in green. The partitions with the bootstrap-by-transfer support higher than 75% are marked with the purple circles. The GenBank identifier for each sequence matches the corresponding sequence row in Fig. 2. Species names are abbreviated; for complete taxonomy, refer to the GenBank entries and discussion in the text. The scale bar for branch lengths represents one substitution per site

We produced a multiple sequence alignment of the representative proteins from many of these clades and inferred a phylogenetic tree of these sequences. Amino acid sequences were aligned using the program MUSCLE v. 3.8.31 [27], and the phylogenetic trees were constructed using the PhyML maximum likelihood approach [38] available through the Booster server at Pasteur Institute (booster.pasteur.fr) and the Galaxy FastTree workflow [72]. The validity of the tree partitions was assessed bootstrap-by-transfer approach with 200 replicates, which has been reported to have higher resolution than the traditional bootstrap and to induce fewer falsely supported branches [65]. The sequence alignment is shown in Fig. 2, the tree is shown in Fig. 3, and the source phylogeny in the Newick format is available as Supplemental Data Set 1.

The phylogeny that we obtained is dominated by a deep split between prokaryotic and eukaryotic sequences; the long internal branch separating those two groups is broken only by two eukaryotic outliers, a fast-evolving homolog from *C.elegans* and a sequence from green alga *Chlorella sorokiniana*. Within eukaryotes, plant FT/CETS homologs segregate as one clade, and fungal/metazoan homologs form another assembly, occasionally intermingled with the representatives of unicellular eukaryotes. A well-defined clade of fungal and metazoan proteins includes FT/PEBP homologs detected recently in an unexpected biological context, namely as the constituents of mitochondrial ribosomal large subunit, where they are known as mitochondrial ribosomal proteins L35/L38. These FT/PEBP/YbhB homologs have distinct N-terminal sequence extensions, found in one gene product within each completely sequenced animal, fungal and protist genome (many of animal and fungal genomes also include additional homologs that do not contain such an extension). This domain is not included in the alignment in Fig. 2; none of the plant FT homologs appear to contain such a region.

The six well-known paralogs in *A.thaliana* are FLOWERING LOCUS T (FT; AT1G65480), TWIN SISTER OF FT (TSF AT4G20370), TERMINAL FLOWER 1 (TFL1; AT5G03840), BROTHER OF FT AND TFL1 (BFT; AT5G62040), MOTHER OF FT AND TFL1 (MFT; AT1G18100), and *Arabidopsis Thaliana* CENTRORADIALIS (ATC; AT2G27550). These sequences, and their counterparts in other species, show the expected topology, with the MFT branch splitting off the common angiosperm stem first, and the FT/TFL branch diversifying later, with additional variations within angiosperms [14, 22, 51, 117]. The sequences from more primitive plants included in the alignment, i.e., moss *Physcomitrium patiens* (former *Physcomitrella*, recently re-absorbed into a larger genus – [75]) and another representative from *Chlorella sorokiniana*, predictably form their own deep clades near the common root of Viridiplantae. Evolution and functional specialization of FT homologs in different lineages of Viridiplantae, including early branching ones, have been extensively reviewed elsewhere [41, 57, 70].

Within the prokaryotic partition of the tree, the phylogeny is less well-resolved; it includes many deep-branching clades, which are often supported statistically but their position relative to other such clades is unclear. As a rule, however, these clades do not mix bacterial and archaeal representatives. On the other hand, on several occasions a group of homologs from closely related bacterial species would include also a sequence from a phylogenetically distant bacterium; in just one example, a sequence from Gram-negative proteobacterium *Helicobater pylori*, WP000846523.1, resides within a group of YbhB-like sequences from Gram-positive bacteria (Fig. 3). This indicates that the evolution of the YbhB family in bacteria must have included occasional horizontal gene transfer events.

The trend discussed above, i.e., the partitioning of all FT/YbhB homologs into the eukaryotic and prokaryotic tribes in the tree, is broken in the case concerning a neglected, uncharacterized member of the FT family in arabidopsis, AT5G01300. The expression of this poorly studied protein in plants and its apparent close similarity to bacterial YbhB may have been first discussed by Schieffer [98], and the expression of an orthologous gene in *Brassica*, as well as its large evolutionary distance from the other homologs, were recently noted in Sheng et al. [102]. In our tree, the sequence of AT5G01300, together with its homologs from another angiosperm and a moss, were clearly nested, with strong statistical support, within the prokaryotic portion (Fig. 3). Database searches and preliminary sequence analysis indicate that nearly all sequenced plant genomes encode such bacteria-like orthologs of AT5G01300 (unpublished data). We propose to call this 7th member of the FT family in Arabidopsis *PYBHB* (for plant YbhB homolog). A more detailed analysis of the phylogenetic origin of PYBHB would require a denser sampling of green plant lineages, as well as of their immediate algal ancestors and bacteria; such an analysis would be of great interest but is beyond the scope of this work.

Taken together, these data suggest, first and forеmost, that FT/CETS/PEBP/RKIP/YbhB-like proteins are ubiquitous throughout the evolution of life, and that nearly all eukaryotic homologs are likely to have a single origin in a prokaryotic ancestor, with a possible secondary retention of a bacterial-like PYBHB clade in plants. Moreover, nearly-universal sequence conservation of several key amino acid residues and the common structural scaffold of the entire protein family (Figs. 1 and 2, and see the next section) suggest that most of those proteins share an ancient conserved molecular function.

#### Sequence-structure analysis of PEBP/RKIP/FT/YbhB proteins supports the hypothesis of a universally conserved function

A common biochemical function of the entire PEBP/YbhB protein family is further suggested by delineation of the conserved and variable sequence elements of the family (Fig. 2). As has been noticed before [11, 12, 99, 100], there are five nearly-invariant polar amino acid residues in the alignment. Several additional highly conserved residues, in particular prolines, are found around these signature amino acids (Figs. 1 and 2). Evolutionary substitutions in the five polar residues are exceedingly rare. Interestingly, multiple replacements in those five sites are observed in just two groups of the PEBP/RKIP/FT/YbhB proteins. One of such groups is the clade of animal and fungal mitochondrial ribosomal proteins L35/L38, which must have acquired a new molecular function during evolution. The other group comprises the plant PYBHB proteins mentioned above. It is tempting to speculate that the PYBHB functions are likewise derived, possibly plant-specific, and could be related to the organelle function; PYBHB sequences, however, do not bear obvious signals for sorting to chloroplasts or mitochondria, and do not seem to cluster with the groups of bacteria that are close to the putative bacterial ancestors of plant organelles (unpublished data).

High-resolution spatial structures of many FT/PEBP/RKIP/FT/YbhB homologs from many diverse species have been obtained (see references above). Mapping the conserved sequence features onto these structures suggests that they are clustered in space, with three of the five most conserved residues located around the rim of the main cavity on the surface of the molecule, and the remaining two forming a salt bridge just above this rim (Fig. 1). The polar hole on the surface of the PEBP/RKIP/FT/YbhB protein molecules binds some of the polar ligands added to the crystallization media, such as cacodylate, phosphotyrosine, phosphoethanolamine, or (4S)-3-[(E)-1-Oxo-2-butenyl]-4-(phenylmethyl)-2-oxazolidinone, a small molecule that disrupts mammalian RKIP interaction with protein kinase Raf. On the other hand, when phosphatidylethanolamine or other polar lipids were included in the media, they were not seen in the electron density. For example, a recent report that FT from arabidopsis may bind phosphatidylcholine (PC) more strongly and more specifically than other lipids [81] has prompted the analysis of high-resolution structures of FT crystallized in the presence of PC. The crystals were obtained in a variety of conditions, but PC molecule could not be located in any of them; computational docking tentatively suggested four binding sites for PC, all located far from the conserved polar cavity [81, 82]. It is also notable that the electrostatic calculations suggest that the overall charge of the cavity, at least in vacuum, is strongly negative, making it perhaps not conducive for direct interactions with phosphate, whereas the charge on the “shoulder” may be more positive (Fig. 1).

A phenotyping experiment on a panel of mutagenized versions of FT [42] has revealed a differential effect of substitutions in the five conserved charged residues. Out of 14 missense mutations in these residues, six did not alter the early-flowering phenotype of the positive control – the wild-type plant in which the FT transgene was overexpressed – and eight caused a dominant-negative effect of flowering delay compared to the wild type (Supplementary Fig. 4 in [42]). Altering a charge in one or more of residues D71, D73, H118 and R119 appears to be particularly effective in switching the phenotype from early to late flowering. This clearly suggests that the charge distribution on the surface of the FT protein, at the cavity rim itself as well as on its adjoining shoulder, is important; even so, we have no mechanistic explanation for the role of those most prominently conserved elements in carrying out the function of FT and its homologs, either in plants or in other species.

In this survey, we are interested whether these signature elements of sequence and structure may suggest a biochemical function of the FT/YbhB homologs. The Mechanism and Catalytic Site Atlas (M-CSA) resource contains annotated information about the amino acid determinants of catalysis in conserved enzyme families [91]. A web interface allows users to specify a set of conserved residues anywhere in the molecule and search the database with such a signature. We queried M-CSA and identified 21 families of enzymes that have two His, two Asp and one Arg in their active centers. These families represent all 7 of the top-level Enzyme Classification classes of activities, i.e., oxidoreductases, transferases, hydrolases, lyases, isomerases, ligases and translocases. If the selection criteria are relaxed to only two histidines and one aspartic acid surrounding the hydrophilic hole, the search retrieves 105 families, corresponding to almost 11% of the 964 entries in the database (the searches can be reproduced at https://www.ebi.ac.uk/thornton-srv/m-csa/browse/ by selecting the set of conserved residues). The specific three-dimensional configuration of these residues in PEBP/RKIP/FT/YbhB proteins appears to be quite unique, however, as judged by the analysis with the PINTS program, which compares similar spatial arrangements of key residues in non-homologous proteins [104]. Nonetheless, it is clear that a combination of two histidines, two aspartates and an arginine has been repeatedly utilized in evolution to build enzymatic active centers, enabling many kinds of catalytic conversions.

These observations are in sharp contrast to what is known about patterns of sequence conservation in non-catalytic ligand-binding protein domains. We collected all domains in PFAM 33.1 [28], using keywords “ligand+bind” and “phosphate-binding”, resulting in 1044 conserved domains. Removal of the clearly annotated enzymatic domains that bind phosphate in their active centers, such as the protein kinases or ATPases, and selection of a non-redundant set of sequence families produces 651 putative non-catalytic ligand-binding domains. For each of those domains, we downloaded the curated seed alignment from PFAM and used the Skylign server [116] to build HMM profiles and to get the information count for the residues of interest. We recorded the identity of all charged residues that were characterized by the information content of more than 50% of the maximum possible value for their position, and found only 25 domains that had more than two conserved charged residues; none of those domains simultaneously had two conserved histidines and a conserved aspartate (Supplemental Data Set 2). Thus, the known conserved ligand-binding domains do not utilize the combination of two histidines and an aspartic acid residue for their non-catalytic interactions with small molecules. Taken together, these investigations of the conserved features in PEBP/FT/YbhB proteins suggest that they are more likely to be a part of the catalytic center than to serve solely as a binding interface.

#### Genomic context of FT homologs suggests connections with small molecule metabolism

We took advantage of the broad taxonomic distribution of the YbhB homologs and tried to infer the putative functional linkages for these gene products on the basis of their genome context (Table 1). The first kind of linkage we studied was the information on domain fusions.

**Table 1:** Genome-context information suggesting functional links between YbhB homologs and various enzymes of biosynthesis and salvage of small molecules

Type of contextual evidence	Functionally linked proteins / domains / COGs	Taxa in which these functions are putatively linked	COG functional categories / Most relevant GO terms // Additional comments
Domain fusions	Glucose-sorbosone dehydrogenase, (Pectate Lyase-like carbohydrate-binding module, FN3-like domain) (COG1881, COG2133)	*Actinobacteria, Gammaproteobacteria*	Carbohydrate transport and metabolism / Hydrolase activity, acting on glycosyl bonds
Operons	Dialkylmaleic anhydride synthesis and conjugation module of tautomycin/tautomycetin biosynthesis operon	*Actinobacteria*	// Five-carbon substrate (a pentose?)
Phyletic vectors (prokaryotes)	Acyl-CoA synthetase, NDP forming (COG1042).	220 species	Energy production and conversion / ATP-binding, N-acetyltransferase activity // Nucleoside-containg substrate and product
Phyletic vectors (prokaryotes)	YbaR/Trm112 activator of RNA and protein methyltransferases; COG2835	233 species	Translation, ribosomal structure and genesis // RNA modification
Phyletic vectors (eukaryotes)	NUDT3 and other NUDIX hydrolases	*Mammalia*	// Hydrolases preferring pyrophosphate-containing substrates (nucleoside phosphates or phospholipids)
Shared putative regulatory motifs	RlmA 23S rRNA m(1)G745 methyltransferase (COG2226)	*Enterobacteriaceae*	Coenzyme transport and metabolism / rRNA base methylation
Shared putative regulatory motifs	YobB putative carbon-nitrogen hydrolase family protein (COG0388)	*Enterobacteriaceae*	Energy production and conversion / Nitrogen compound metabolic process
Shared putative regulatory motifs	AdrB c-di-GMP phosphodiesterase (COG2200)	*Enterobacteriaceae*	Signal transduction mechanisms / Cellular response to DNA damage stimulus // Nucleoside substrate
Integrated evidence	RlhA 23S rRNA 5-hydroxycytidine C2501 synthase (COG0826)	n/a	Signal transduction mechanisms / rRNA processing, rRNA modification

See text for a more detailed characterization of each putative functional link

It is quite common for two protein domains to exist as separate genes in some species, but to be fused into one gene encoding a multidomain protein in others. Such translational fusions, especially those that are evolutionary conserved, are strongly enriched in proteins that work in concert with one another [45, 105]. Analysis of YbhB homologs in bacteria and archaea shows that they are most commonly encoded as stand-alone open reading frames and form domain fusions infrequently. In actinobacteria, however, the YbhB homologs are frequently found as the C-terminal portions of longer proteins, fused to the modules implicated in carbohydrate metabolism. For example, protein WP_014179790.1 in *Streptomyces* sp. consists of the N-terminal pectate lyase-like carbohydrate-binding module, followed by the FN3 repeat region, a putative glucose/sorbosone dehydrogenase (GSDH) region with predicted beta-propeller structure, and finally the C-terminal YbhB homology domain. This theme is partially preserved, albeit with domain rearrangement, in some species of evolutionarily distant proteobacteria, where the order of domains is GSDH-YbhB, or occasionally GSDH-FN3-YbhB; examples include WP_014747134.1 in an alphaproteobacterium *Tistrella* and proteins in gammaproteobacteria, such as WP_096298086.1 in *Luteimonas*, PYD93448.1 in *Pseudomonas syringae* pv. *pisi*, or WP_145513070.1 in *Xantomonas perforans*. GSDH enzymes utilize quinone cofactors to convert hexoses and their derivatives into a variety of products in bacteria [79], and it is conceivable that the C-terminal YbhB homology domains are involved in the transformations of GSDH substrates or products (or, possibly, in the metabolism of its cofactor).

We then surveyed the databases of conserved genomic neighborhoods and scanned the literature for the evidence of conservation of FT/YbhB chromosomal neighbors in different genomes. As with protein fusions, the FT/YbhB family genes are rarely found within conserved positional associations, such as operons, with other ORFs. In two species of *Streptomyces*, however, YbhB homologs are located within large (20 genes) operons responsible for biosynthesis of polyketide-based small molecules, i.e., tautomycin in *S.spiroverticillatus* and tautomycetin in *S.griseochromogenes*. These polyketides are of pharmacological interest, because they are potent inhibitors of mammalian protein phosphatases. The *ybhB* family genes, called, respectively, *ttnL* and *ttmL*, together with the adjacent seven open reading frames, form a subsystem that is required to manufacture a rare dialkylmaleic anhydride moiety of tautomycin and tautomycetin and to conjugate it to the polyketide backbone, produced by the remaining genes in the same operon. Dialkylmaleic anhydride is required for the activity of the mature product, and is made de novo from propionate and an unidentified five-carbon compound, through the succession of the steps that are incompletely understood [66, 67]; it is plausible that the products of *ttnL* and *ttmL* play a role in the utilization of such a compound – perhaps a sugar or its derivative.

Another approach of connecting genes into functionally related groups is to analyze their phyletic vectors, i.e., the representations of the presences and absences of their orthologous genes in different genomes [33, 35]. We analyzed phyletic vectors using the psi-square program [34] with default settings and vector similarity measured using Pearson correlation-based distance. The vector of YbhB (COG1881) was used as the query, and the NCBI COG database release of 2014 was used as the search space (digitized phyletic vector information was not available for the 2021 COGs release at the time of writing). After retaining the matching vectors of comparable cardinality, i.e., the genes present in 300–400 species, to avoid spurious matching to nearly-ubiquitous proteins, we found two COGs that were most often co-inherited by the same genomes as COG1881. One of those, COG1042, is annotated as Acyl-CoA synthetase (NDP forming); it is found in 358 species, 220 of which encode both COG1881 and COG1042. The enzyme, best studied in hyperthermophilic archaea and protists, is involved in the substrate-level phosphorylation, by the equation acetyl-CoA + ADP + Pi ⇌ acetate + ATP + CoA [80]. The roles of the bacterial homologs of this enzyme is less clear, as some of them appear to be catalytically inactive and possibly play auxiliary roles in the acylation and deacylation of proteins [111]. The other gene with matching phyletic pattern, COG2835, annotated as “uncharacterized conserved protein YbaR, Trm112 family”, is found in 390 genomes, 233 of which also encode COG1881. YbaR/Trm112 family encodes activators of several methyltransferases involved in modification of rRNA, tRNA and peptide release factors [113].

One more way to predict functional linkages between gene products in bacteria is to examine their putative regulatory regions, which tend to be located in the intergenic spacers, usually to the 5′ ends of the open reading frames or operons they regulate. We performed an analysis of conserved upstream regions of *ybhB* genes, using the complete bacterial genomes extracted from Ensembl Bacteria database (release 47) [43]. The regulatory regions of homologous genes were aligned with the MUSCLE program, the most conservative regions were chosen to build a positional weight matrix by the SignalX routine of the Genome Explorer program [78], and the genomes were scanned by Genome Explorer with the threshold equal to the lowest score in the training set, i.e., in the *ybhB* homologous regions. Only some species in the family *Enterobacteriaceae* had a detectable conserved site upstream of the y*bhB* open reading frame. Unlike most known binding sites for the specialized transcription factors, this site lacked palindromic structure and has a consensus sequence TACACTT. Scanning 41 *Enterobacteriaceae* species from 14 genera with a probabilistic model of the site, we identified additional intergenic regions where the variants of these sites occur (Table 1, and Supplemental Data Set 3). Three genes were found to contain a highly similar conserved upstream site in 16–17 species, representing 8 genera; these were *rlmA* (23S rRNA m(1)G745 methyltransferase), *yobB* (putative carbon-nitrogen hydrolase family protein), and *adrB* (c-di-GMP phosphodiesterase). Sites matching this consensus in *E.coli* have been noticed before and hypothesized to represent a modified form of the canonical -10 element sequence TATAATT [44], though the regions in which we found this upstream element do not appear to have the recognizable -35 sequence nearby. The significance of the shared TACACTT element thus remains unclear, though a common regulation mechanism for genes that share this site is a possibility.

We also have consulted several databases of gene essentiality, as well as curated databases of pre-computed functional linkages between genes in various model organisms. The bacterial fitness database [88] reports a moderate reduction in mobility for an *E.coli* mutant with transposon-tagged YbcL, a prophage-encoded paralog of YbhB, whereas the Database of Essential Genes [71] identifies the YbhB counterpart in *H.pylori* as essential; in neither of this cases is there any information about possible molecular mechanisms. The FunCoup database, which uses naïve Bayesian approach to integrate information on interactions and functions from 10 different genomic and proteomic measurement spaces [85], shows a small network of interacting proteins in *E.coli*, consisting of two chaperones, i.e., a heat shock 70-family DnaK and a protease Lon, as well as YbhB and RlhA. Interactions with chaperones are frequently observed in the protein interaction data and may reflect non-specific cellular proteostasis needs and a broad clientele of many chaperone systems; the connection to the other protein in the group, RhlA, may be more revealing, as it appears to be a component of the 5-hydroxycytidine synthase enzymatic complex, involved in modification of 23S rRNA [53]. Finally, the analysis of phyletic patterns across many eukaryotes, collected in PhyloGene database [96] has revealed one highly-scoring match co-inherited with human PEBP1 gene, i.e., NUDT3, encoding an enzyme from the Nudix class. Nudix proteins are hydrolases noted for the affinity to the pyrophosphate moieties in their substrates, often lipid, nucleoside or oligonucleotide derivatives [74].

## Conclusions

“The burden of proof should be proportional to the strangeness of the facts.”

George Flournoy. Translated from *Des Indes à la Planète Mars: Étude sur un Cas de Somnambulisme Avec Glossolalie.*

“Circumstantial evidence is a very tricky thing,” answered Holmes thoughtfully. “It may seem to point very straight to one thing, but if you shift your own point of view a little, you may find it pointing in an equally uncompromising manner to something entirely different.”

Arthur Conan Doyle. *The Boscombe Valley Mystery*.

In this survey, we reviewed, by necessity briefly, the strong genetic evidence of the key role of the FT/CETS family in the floral induction in plants. We also argue that, while the evidence of genetic interactions of *FT* gene with other genes in the floral induction pathways is not in doubt, the molecular function of the FT proteins in flowering may have been misunderstood. Indeed, the analysis of literature, as well as many orthogonal computational experiments presented here, suggest that the proteins from the FT/CETS/PEBP/RKIP/YbhB family, which are ubiquitous not only in plants, but also in animals, fungi, protists, prokaryotes and giant viruses, might be catalytic subunits of the enzymes involved in biochemical transformations of small molecules, in addition to, or even instead of, their postulated role of being stoichiometric subunits within plant transcription complexes.

Much of the analysis reported here comes from the examination of prokaryotic genome sequences. Obviously, studies in archaea and bacteria cannot be expected to validate the role of FT as a transcriptional co-activator promoting floral induction, as prokaryotes lack most of the plant signal transduction systems and downstream effectors of flowering. Instead, we asked two different questions, i.e., “What can be deduced about the molecular functions of the FT orthologs in prokaryotes?” and “Is there any evidence that these functions may be conserved in eukaryotes, including plants?”

The first of these questions can be tentatively answered by examination of the genomic context of FT/YbhB homologs. Though no single gene could be repeatedly linked to YbhB by multiple approaches, a trend emerges when the genomic context data are considered jointly (Table 1). The evidence appears to point towards functional linkages of YbhB to sugar and/or ribonucleoside modifications, implying that FT/YbhB may be involved in the metabolism of a monosaccharide such as ribose or another pentose, or perhaps their nucleotide-like derivative. Relatedly, FT proteins could be involved in phospholipid metabolic pathways, which include lipid-nucleotide conjugates as key intermediates [48].

The answer to the other question posed above, i.e., whether the putative molecular function have been preserved throughout the evolution of the FT/CETS/PEBP/RKIP/YbhB family, appears to be clearly “yes”. There is a striking pattern of sequence conservation and spatial juxtaposition of the key charged residues in the family at a long phylogenetic span (Figs. 1 and 2), with only two narrow clades experiencing major disruption in those positions (Figs. 2 and 3). One of those clades comprises proteins with a changed function (mitochondrial ribosomal components), and the function of the other, which includes a divergent FT homolog, is enigmatic.

Bioinformatic analysis suggests that the conserved sets of two histidines and an aspartic acid clustered in space are frequent in the enzymatic active centers but are not found in the non-catalytic ligand-binding domains. In the same vein, broad occurrence of a protein family in bacteria, archaea, giant viruses and nearly all eukaryotes may not be unusual for a metabolic enzyme, but would be a rare occurrence for transcriptional regulators, as they are typically not shared between bacteria and eukaryotes [9].

Several recent observations on the role of sugar and lipid metabolism genes in floral induction may be relevant. For example, StSP6A protein, the potato ortholog of FT, is co-expressed with a sugar transporter SWEET11 in the stolon apical and sub-apical meristems during tuber induction, and the products of two proteins interact physically in a heterologous split-ubiquitin assay in yeast cells [3]. In аrabidopsis, a related sugar transporter, SWEET10, appears to be regulated by FT at the transcriptional level, though the data on physical interactions in that case have not been reported [7]. Also, manipulations of the biosynthetic enzymes producing trehalose-6-phosphate in аrabidopsis have shown that floral response and shoot branching require this phosphosugar, which apparently acts through the FT pathway [30, 114]. Genetic evidence has also suggested the involvement of phosphorylethanolamine cytidylyltransferase (*PECT1* gene product) in flowering in аrabidopsis [106].

There is an ample precedent of utilization of sugars, nucleotides and products of RNA breakdown for plant hormone biosynthesis. One class of plant hormones, cytokinins, are purine derivatives that can be produced either by isoprenylation of adenosine phosphate or by tRNA degradation [50], whereas another class, gibberellins, are synthesized by transforming the pentose skeleton generated in the 1-deoxy-D-xylulose 5-phosphate pathway [97]. Recent studies have significantly expanded the repertoire of linear and cyclic oligonucleotides that serve as essential signaling messengers in bacteria and animals [17, 18], suggesting that some nucleoside derivatives with regulatory properties may remain undiscovered in plants.

A critic might point out that bioinformatic evidence of the enzymatic function of FT and their homologs presented in this manuscript is too circumstantial – some would say, strange and/or speculative – and that it has not been corroborated by direct wet-laboratory experiments. However, exactly the same can be said about the experimental wet-lab support for the role of FT as a transcriptional co-activator – a hypothesis that is based on several lines of suggestive evidence, but is unmoored from the hard facts of comparative genomics. In the spirit of considering the entire corpus of the available data, and using reciprocal illumination from different classes of evidence to improve the precision of scientific hypotheses [10, 20, 46], it seems timely and urgent to test whether the FT homologs in various organisms may have an enzymatic activity.

One experimental approach would be to compare small-molecule metabolite profiles in the *ybhB* knockout mutants and identify compounds that are in a shortage or excess in the mutant compared to the wild type. Bacterial model systems may be perhaps most amenable to such an analysis, as *ybhB* homologs tend to be single-copy in bacteria and are dispensable for growth in laboratory culture in most tested strains. Metabolomics-based identification of putative products or substrates of YbhB homologs would be the first step in determining the putative enzymatic activity that we predict to be conserved in most members of this protein family, and may lead the way to the identification of the small-molecule component of the plant flowering hormone.

## Supplementary Information

**Additional file 1: Supplemental Data Set 1.** Newick-formatted phylogenetic tree of FT/YbhB homologs in bacteria, archaea and eukaryotes.

**Additional file 2: Supplemental Data Set 2.** List of ligand-binding domains in PFAM and their usage of conserved amino acid residues.

**Additional file 3: Supplemental Data Set 3.** Occurrences of the motif with consensus sequence TACACTT in *Enterobacteriaceae.*

## Data Availability

The datasets supporting the conclusions of this article are included within the article and its additional files.

## Abbreviations

COG: Clusters of Orthologous Groups
GSDH: glucose/sorbosone dehydrogenase
PC: phosphatidylcholine
PEBP: phosphatidylethanolamine-binding protein
RKIP: Raf-1 kinase-inhibiting protein
SAM: shoot apical meristems
